# Barriers to Treatment Access for Chagas Disease in Mexico

**DOI:** 10.1371/journal.pntd.0002488

**Published:** 2013-10-17

**Authors:** Jennifer M. Manne, Callae S. Snively, Janine M. Ramsey, Marco Ocampo Salgado, Till Bärnighausen, Michael R. Reich

**Affiliations:** 1 Department of Global Health and Population, Harvard School of Public Health, Boston, Massachusetts, United States of America; 2 Department of Health Policy and Management, Harvard School of Public Health, Boston, Massachusetts, United States of America; 3 Regional Center for Public Health Research, National Institute for Public Health, Tapachula, Mexico; 4 State of Morelos Secretary of Health, Program on Chagas Disease, Cuernavaca, Mexico; Emory University, United States of America

## Abstract

**Background:**

According to World Health Organization (WHO) prevalence estimates, 1.1 million people in Mexico are infected with *Trypanosoma cruzi*, the etiologic agent of Chagas disease (CD). However, limited information is available about access to antitrypanosomal treatment. This study assesses the extent of access in Mexico, analyzes the barriers to access, and suggests strategies to overcome them.

**Methods and Findings:**

Semi-structured in-depth interviews were conducted with 18 key informants and policymakers at the national level in Mexico. Data on CD cases, relevant policy documents and interview data were analyzed using the Flagship Framework for Pharmaceutical Policy Reform policy interventions: regulation, financing, payment, organization, and persuasion. Data showed that 3,013 cases were registered nationally from 2007–2011, representing 0.41% of total expected cases based on Mexico's national prevalence estimate. In four of five years, new registered cases were below national targets by 11–36%. Of 1,329 cases registered nationally in 2010–2011, 834 received treatment, 120 were pending treatment as of January 2012, and the treatment status of 375 was unknown. The analysis revealed that the national program mainly coordinated donation of nifurtimox and that important obstacles to access include the exclusion of antitrypanosomal medicines from the national formulary (regulation), historical exclusion of CD from the social insurance package (organization), absence of national clinical guidelines (organization), and limited provider awareness (persuasion).

**Conclusions:**

Efforts to treat CD in Mexico indicate an increased commitment to addressing this disease. Access to treatment could be advanced by improving the importation process for antitrypanosomal medicines and adding them to the national formulary, increasing education for healthcare providers, and strengthening clinical guidelines. These recommendations have important implications for other countries in the region with similar problems in access to treatment for CD.

## Author Summary

Chagas disease is a vector-borne disease caused by the parasite *Trypanosoma cruzi*. The disease is most frequently transmitted by triatomine insects but can also be passed through blood donation or from mother to child at birth. Experts estimate that 8 million people are infected with Chagas disease globally and that 1.1 million of these infections are found in Mexico. Most public health programs for Chagas disease focus on preventing new infections through vector control and screening the blood supply. However, in recent years there has been a greater focus on treating the disease with one of two available medications, benznidazole or nifurtimox. This study explores access to these two drugs in Mexico. The study shows that less than 0.5% of those who are infected with the disease received treatment in Mexico in years. The study also identified important factors that limit access in Mexico, including the exclusion of both drugs from the national health insurance program and problems importing these medications. Finally, the paper suggests ways that these problems can be overcome in Mexico, while providing helpful insight for other countries that struggle with similar problems in treating this disease.

## Introduction

Chagas disease is a vector-borne, parasitic disease with a prevalence of 8 million infections globally. The disease is responsible for as many as 15,000 deaths per year [1], [2], largely concentrated among the poor in Latin America, and a recent study found that the disease is also responsible for substantial losses in productivity and a large economic burden, especially in high prevalence countries [3]. *Trypanosoma cruzi*, the etiologic agent of Chagas disease, is most often transmitted by contact with infected triatomine insects, though transmission can also occur congenitally and through blood transfusion or organ transplantation [4]. In 2009, it was estimated that less than 1% of those infected with *T. cruzi* received treatment for the disease globally [5].

According to prevalence estimates for 2006 from the World Health Organization [6], approximately 1.1 million people are infected with *T. cruzi* in Mexico. However, limited published information exists on how many patients receive treatment in Mexico and what obstacles may hinder access to treatment. This study sought to determine for Mexico: (1) the extent of treatment access for Chagas disease; (2) the national level barriers to access to treatment for Chagas; and (3) strategies that could be used to overcome these barriers and increase access to treatment for Chagas disease.

This study uses an existing health systems framework, the Flagship Framework for Pharmaceutical Policy Reform [7], to analyze the barriers to treatment access for Chagas disease in terms of five policy interventions – regulation, financing, payment, organization, and persuasion. Based on this analysis, we also suggest strategies to increase access.

### Diagnosis and treatment of Chagas disease

Chagas disease is clinically manifested in two stages – an acute stage and a chronic stage. The acute stage lasts for approximately 4–8 weeks and is characterized by flu-like symptoms or a characteristic local swelling at the site of parasite entry [8], [9], following which an infected person enters the indeterminate form of the chronic phase of infection. Among those with the indeterminate chronic form, about 20–30% of patients progress to the chronic cardiac or digestive forms of Chagas disease [10]. The most common course of Chagasic cardiomyopathy includes conduction system abnormalities early in the disease, resulting in heart failure. In all phases, serological tests such as the enzyme-linked immunosorbent assay (ELISA) test, the indirect haemagglutination assay (IHA), and the indirect immunofluorescent antibody test (IIF) are used for diagnosis [4], [9], [11]. Because these tests can be difficult to interpret, the WHO recommends the use of two concomitantly positive tests to make a confirmed diagnosis [11], [12].

Currently, benznidazole and nifurtimox are the only antitrypanosomal medicines available to treat *T. cruzi* infection. Antitrypanosomal therapy is strongly recommended by WHO for acute, congenital or reactivated infections, and for chronic infection in children under the age of 18 [13], [14], [15]. Recent scientific evidence about the clinical effectiveness of these medications has led to the expansion of treatment indications to include adults in the chronic phase of the disease without advanced cardiomyopathy [1], [11], [16], [17], [18], [19]. Though no randomized controlled trial has directly compared the two medications [11], WHO guidance and the clinical literature place greater emphasis on the use of benznidazole [4] as a first-line therapy because there is more clinical evidence for its efficacy, and it has a more favorable side-effect profile and is better tolerated by adult patients [9], [15], [16], [17], [18], [20]. A randomized clinical trial of benznidazole is underway to determine its efficacy in slowing progression of disease among patients with early to moderate stage Chagasic cardiomyopathy [21], [22].

Both benznidazole and nifurtimox have undergone changes to their global supply chains over the past decade. Benznidazole was manufactured by Roche until 2003, at which time the rights and manufacturing technology were transferred to the Pernambuco state pharmaceutical laboratory in Brazil, Laboratorio Farmaceutico do Estado Pernambuco (LaFepe) [23], [24]. Between 2004 and 2006, LaFepe produced several batches of benznidazole using active pharmaceutical ingredient that was donated by Roche [24]. Then, after a period of no production, LaFepe resumed production of benznidazole in late 2011 and the medicine is now distributed by several entities including LaFepe, WHO, and Masters Pharmaceuticals. Nifurtimox is manufactured by Bayer HealthCare in El Salvador. In 2007 Bayer reached an agreement with WHO for Bayer to donate nifurtimox to WHO and for WHO to distribute the medicine through the WHO-Bayer Nifurtimox Donation Program [25].

### Chagas disease and the Mexican health system

Access to treatment for Chagas disease in Mexico must be considered in the context of the Mexican health system and its recent reforms. Mexico has three major national insurance schemes, the *Instituto Mexicano del Seguro Social* (IMSS), *Instituto de Seguridad y Servicios Sociales de los Trabajadores del Estado* (ISSSTE), and *Seguro Popular* (SP) [26]. IMSS and ISSSTE together offered coverage to approximately 42.6 million private sector (IMSS) and public sector (ISSSTE) employees in 2010 [26]. As of 2011, SP, a social health insurance program started in 2003, offers a package of 284 essential services to approximately 51.8 million Mexicans, according to the Mexican government [10], [27], [28], [29]. Affiliation with SP requires a fixed family contribution that is based on a progressive scale by income, though individuals and families who fall in the lowest two income deciles are exempt from payment of a premium [26], [27].

**Figure 1:**
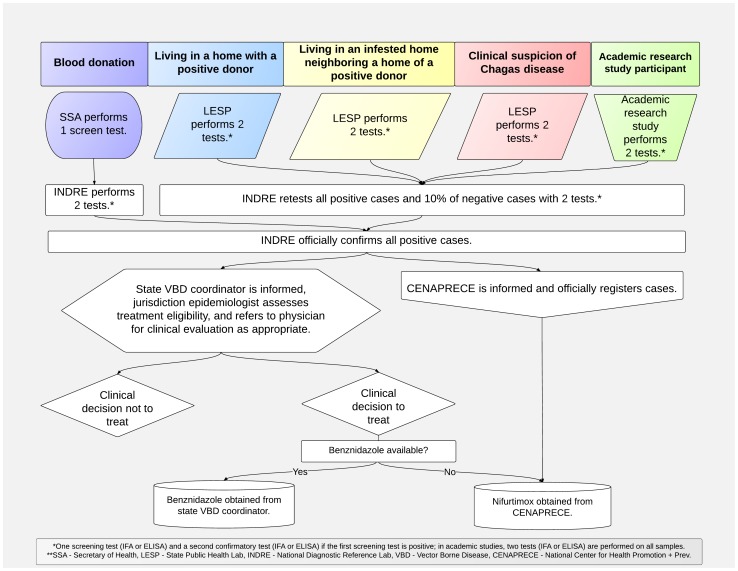
Chagas disease diagnostic and treatment patient flow in Mexico.

The national Program on Onchocerciasis, Leishmaniasis and Chagas Disease within the Mexican Secretary of Health's National Center for the Prevention and Control of Diseases (CENAPRECE) is the unit responsible for establishing guidelines and coordinating national activities for Chagas disease control. The State Secretaries of Health report patients who are diagnosed by ISSSTE, IMSS and SP systems to the national Program, which then turn provides medicines to treat confirmed cases. Figure 1 shows the process of case registration for a patient with Chagas disease.

## Methods

### Ethics statement

IRB exemption was obtained from Harvard School of Public Health (Protocol# 21514-101) and the National Institute for Public Health (INSP) located in Cuernavaca, Mexico. Oral informed consent was obtained from all interviewees.

### Theoretical framework

#### Defining and quantifying access

Access is an important and frequently addressed theme in public health, and multiple frameworks exist to define access and its possible determinants [30], [31]. In this study, we use a general definition of access as “the ability to obtain and appropriately use a good quality health technology when it is needed” [32].

Given that antitrypanosomal medicines are procured based on the number of registered cases, we first quantify the number of cases registered by CENAPRECE over the period of 2007–2011. We then assess the extent of case registration for Chagas disease in the following ways: (1) by comparing the number of cases registered to the expected number of prevalent cases according to the most recent (2010) estimate of Chagas disease prevalence from the Mexican Secretary of Health (733,333 cases, 0.652% prevalence); and (2) by comparing the number of cases registered to the targets for new case registration that were established by the national Program on Onchocerciasis, Leishmaniasis and Chagas Disease within CENAPRECE. We use the most recent estimate from the Secretary of Health because it is the most conservative estimate available. The details of prevalence estimates for Mexico are summarized in Table 1.

**Table 1:** Prevalence estimates and case registration in Mexico.

	National Level
Population	112,340,000
Prevalence Estimate - Blood Bank Estimate	0.652%
Expected Cases - Blood Bank Estimate	733,333
Cases registered (2007–2011)	3,013
% of Target (2007–2011)	80.48%
% of Expected Cases - Blood Bank	0.41%

Next, we examine the treatment status of cases registered in the period 2010–2011 in terms of those who received treatment, those who were pending treatment as of January 2012, and “others” whose treatment trajectory was unknown. We define the gap in access as the number of cases registered in 2010–2011 that were treatment eligible but had not yet received treatment with benznidazole or nifurtimox at the time of the study (January 2012). Due to a lack of data, we were unable to quantify the proportion of registered cases that were treatment eligible and how this compares to the proportion that received treatment. As a sub-analysis, we assess the clinical quality of treatment access for patients; we define clinical quality as the proportion of patients receiving treatment with benznidazole, the first-line medication, as compared to those receiving treatment with nifurtimox, the second-line medication. Although there is no official WHO guideline that defines benznidazole as the first line treatment, the use of benznidazole over nifurtimox is supported by evidence in the literature detailing the clinical effectiveness of benznidazole, its superior side effect profile, and its sole use as the comparator in clinical trials [9], [16], [17], [21], [33]. We therefore adopt benznidazole treatment as our indicator of clinical quality.

#### The Flagship Framework and access to treatment for Chagas disease in Mexico

Given that treatment for Chagas disease is provided in the context of the state and national healthcare system, we sought a framework that could examine access to Chagas medicines in the pharmaceutical sector, could assess key institutions in the health system and their interactions, and could be used to develop strategies for reform.

**Figure 2:**
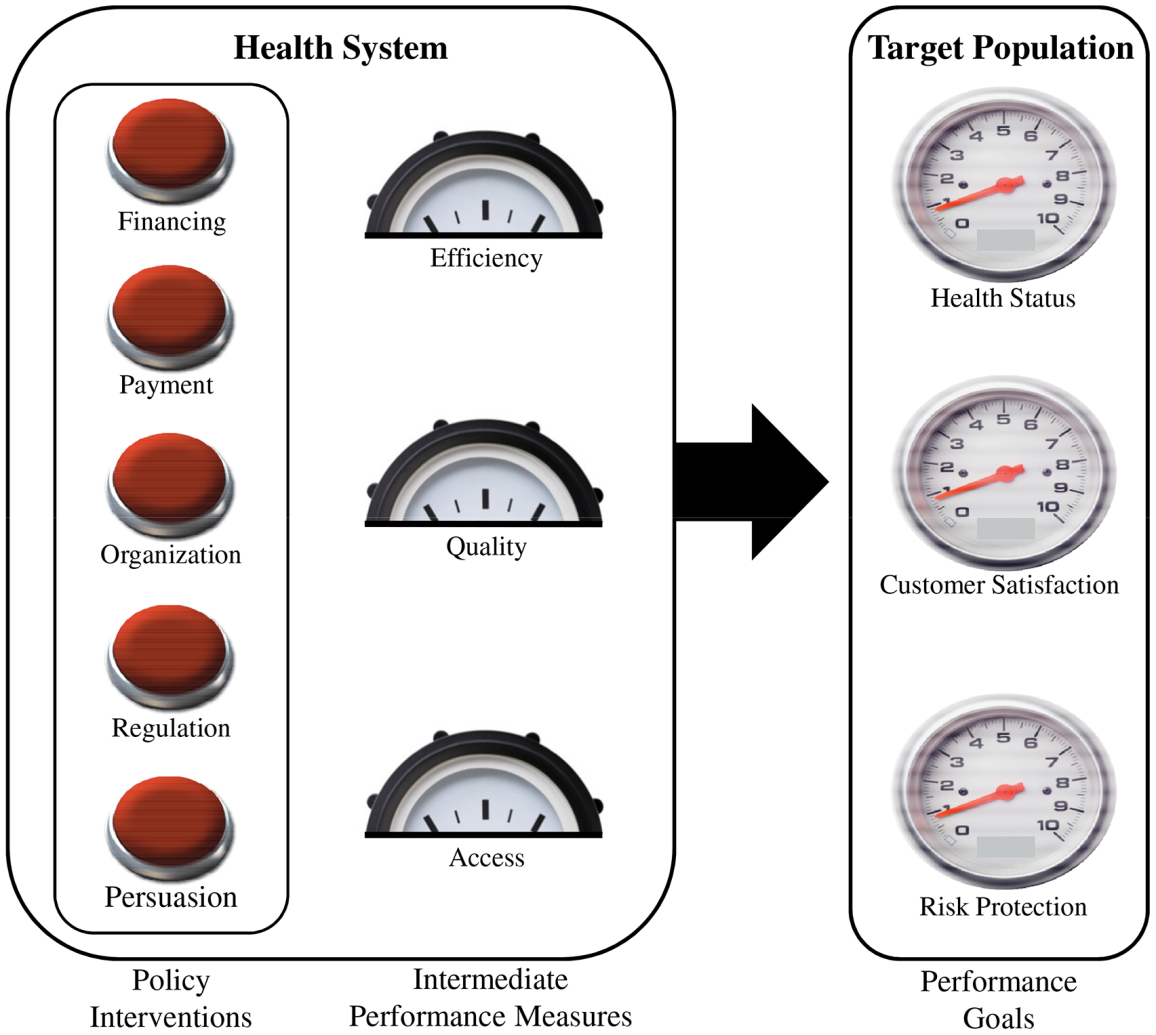
Flagship Framework for Pharmaceutical Policy Reform [7].

We use the Flagship Framework for Pharmaceutical Policy Reform to analyze barriers to treatment access for Chagas disease in Mexico. This Framework presents three ultimate performance goals of the pharmaceutical sector: health status, citizen satisfaction, and financial protection (Figure 2). These goals represent the dependent variables in the framework, affected by five categories of independent variables called the “control knobs” (hereafter policy interventions) of financing, payment, organization, regulation, and persuasion [7]. These policy interventions are linked to the ultimate performance goals through three important characteristics that describe the functioning of various subsystems of the pharmaceutical sector. These three “intermediate performance goals” are efficiency, access, and quality [7].

Our analysis in this paper centers on access, one of the three intermediate performance goals, as the dependent variable of interest; as a sub-analysis, the clinical quality of the treatment received by patients is also partially addressed, according to the definitions provided above. Access to antitrypanosomal treatment as an intermediate performance goal can be linked to the ultimate performance goal of health status via the established relationship between treatment with antitrypanosomal therapy and improvements in the health status of *T. cruzi* infected patients. It has been shown that antitrypanosomal therapy with benznidazole or nifurtimox prevents or slows the progression of chronic Chagas disease and increases quality-adjusted life expectancy [16], [17], [18]. This paper's definition of clinical quality, as the proportion of patients receiving first-line treatment with benznidazole, is related to the ultimate performance goal of health status because there is greater evidence in the literature showing the clinical effectiveness of benznidazole and the fact that it is better tolerated by patients, and thus that it has a greater positive impact on health status. It is important to note that data on treatment adherence or completion, which are also important for clinical quality, were not available for patients who received either medicine.

We organize our analysis of barriers around the five policy interventions as categories of independent variables that affect access. We adopt the definitions for these policy interventions directly from the Flagship Framework. Regulation refers to government efforts to alter behavior in the private and to a lesser extent the public sector by imposing rules that are backed by sanctions. Payment focuses on what and how various organizations and individuals in the pharmaceutical sector are paid and the incentives created by those payments. Financing refers to how the money for pharmaceuticals is raised and how this affects the distribution of use and costs across the infected population. Organization focuses on how activities in the pharmaceutical sector are divided among public and private entities and centralized and decentralized agencies. Persuasion refers to community engagement and efforts to convince specific actors (doctors, patients, policymakers, etc.) to change certain behaviors through education, social marketing, or health communication activities [7]. In this analysis, regulation is discussed first because it affects findings in other policy interventions.

#### Data collection

Guided by the Flagship Framework, we created a list of possible obstacles to treatment access for Chagas disease in Mexico [7], [32]. Based on this initial assessment, we constructed three interview guides: one for key informants, a second for national level actors, and a third for state level actors. We also searched the academic literature and public government websites for national regulations, policies and laws relevant to the topic of Chagas disease treatment in Mexico. This documentation served as a source for triangulation of information obtained in interviews.

Semi-structured in-depth interviews were conducted with 18 key informants and policymakers. Contact with initial interviewees was established through in-country key informants. Subsequent interviews were then obtained through a snowball sampling method, whereby participants in the interviews facilitated contact with other relevant actors [34]. The sampling strategy did not specify a desired number of actors to be interviewed at each level, but relied on the concept of saturation, such that interviews were conducted until responses to the survey questions were repetitious or until all relevant actors had been contacted [34].

Interviews were conducted by one member of the research team (JM) in English or Spanish, depending on the preference of the interviewee, and written notes were taken of interview responses. In all interviews, written documentation of policies, procedures, laws, or governmental permits mentioned by the interviewee were requested if they were not already publicly available.

#### Data analysis

To answer our second research question on the barriers to treatment access, we organized national regulations, policies and laws as well as interview responses into categories corresponding to the policy interventions in the Flagship Framework for Pharmaceutical Policy Reform. Reliance on multiple sources of written and oral information enabled triangulation of the information obtained. This allowed us to minimize bias that could be present in the responses of any one source [35].

## Results

### Access to treatment for Chagas disease at the national level

#### Registration of cases

**Figure 3:**
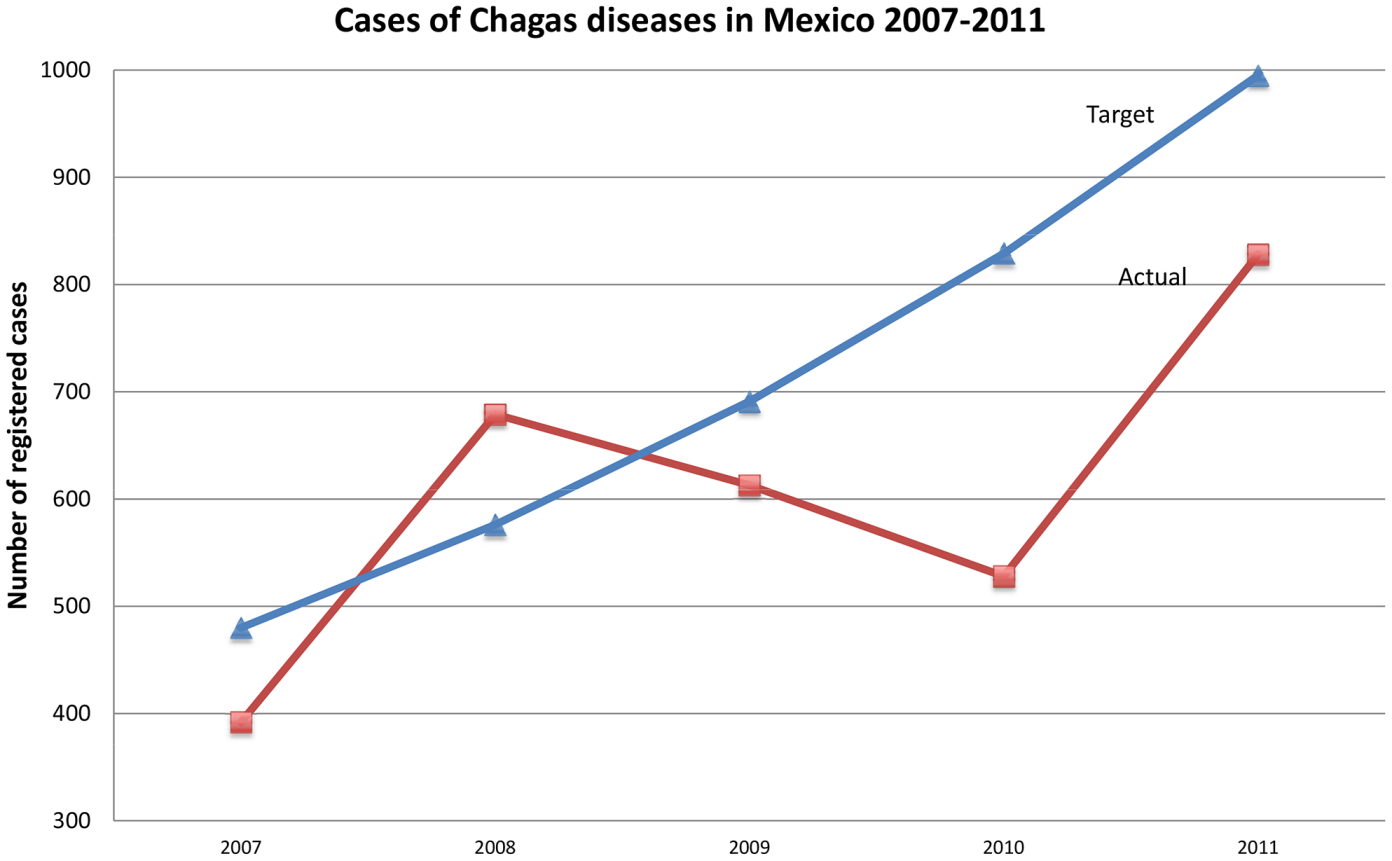
Data on access to treatment for Chagas disease in Mexico, 2007–2011. *Note on* *Figure 3* *: It was assumed that an additional 166 registered cases that the Secretary of Health reported in 2013 had been diagnosed and registered in 2011 but had not yet been confirmed at the time of the initial data provision in January 2012*.

The Secretary of Health in Mexico reports that 3,013 cases of Chagas disease were registered by the national Program on Onchocerciasis, Leishmaniasis and Chagas Disease (hereafter national program) within CENAPRECE from 2007–2011. The number of cases registered represents approximately 0.41% of the total expected cases, according to Mexico's estimates of national prevalence [36], [37]. Moreover, in every year during this period but one (2008), the number of new registered cases was below the national program's target number for new registered cases by 11–36% (Figure 3) [38]. The national targets correspond to a 20% increase in registered cases per year, beginning in 2006. These findings show that the number of new cases registered by the national program is substantially below the number of new registrations expected and did not meet targets for case registration established by the national program.

The vast majority of registered cases (nearly 90%) between 2007 and 2010 were diagnosed and treated by the Secretary of Health. Based on this information, we focus the remainder of our analysis exclusively on SP as this is the insurance program that increasingly provides care to these patients [38].

#### Antitrypanosomal medicine procurement and treatment of cases

Since 2009, the national program has offered medicines for treatment of registered cases at the state level by requesting nifurtimox from the WHO-Bayer Nifurtimox Donation Program. Our study found that procurement initiated in 2007 took more than two years due to an inability of the national program to secure all necessary importation and storage permits within Mexico. Procurement initiated since 2009 has taken 4–6 months. Once nifurtimox is received, the national program then provides it on request to state Secretaries of Health, which in turn distribute the allocated amount to the appropriate providers for the treatment of identified patients [38]. The only exception to this procurement process is that the Morelos state Program on Chagas Disease chose to purchase benznidazole directly from Masters Pharmaceuticals, Ltd. in 2010. This process took approximately one year, with the medicines received in 2011.

Data from the national program indicate that, of the 1,329 cases registered nationally in 2010–2011, 834 (62.7%) of these patients received treatment; in addition, our analysis found that 120 (10.1%) of the cases were pending treatment at the end of 2011 due to an insufficient supply of nifurtimox, and the treatment status of the remaining 375 (28.2%) was unknown [38]. All cases in Mexico reported by the national program were treated with nifurtimox except for those treated in the state of Morelos. No data were available at the national level on the proportion of cases treated prior to 2010–2011 or about the length, dosing and efficacy of treatment provided.

### Analysis of barriers at the national level

Table 2 provides a list of national level obstacles to treatment access for Chagas disease, based on our analysis of data collected in this study. The list includes all obstacles that were mentioned during interviews and could be triangulated using a second data source.

**Table 2:** National level obstacles to treatment for Chagas disease in Mexico, by medicine.

Policy Intervention	Benznidazole	Nifurtimox
Regulation	• Lack of national regulatory body (COFEPRIS) approval	• Lack of national regulatory body (COFEPRIS) approval
	• No commercial license, each importation requires a separate permit	• No commercial license, each importation requires a separate permit
	• Not included in national formulary for Mexico (Cuadro Basico)	• Not included in national formulary for Mexico (Cuadro Basico)
Financing	• Funds unavailable from Secretary of Health or SP to purchase Chagas disease medicines	• Funds unavailable from Secretary of Health or SP to purchase Chagas disease medicines
Payment	• Medicine and donation costs fall to governmental agency	• Medicine available for free, funds available through donation program to support distribution costs
Organization	• Mexican norms for vector borne diseases indicate use for acute and chronic Chagas disease in patients up to 70 years old; name benznidazole second-line therapy	• Mexican norms for vector borne diseases indicate use for acute and chronic Chagas disease in patients up to 70 years old; name nifurtimox first-line therapy
	• No national clinical guidelines for Chagas disease treatment	• No national clinical guidelines for Chagas disease treatment
	• Chagas disease included in SP as of 2012 but drug excluded from SP list (CAUSES)	• Chagas disease included in SP as of 2012 but drug excluded from SP list (CAUSES)
	• Benznidazole not on essential medicines list for Mexico	• Nifurtimox not on essential medicines list for Mexico
	• Global supply chain problems: insufficient global supply	• Global supply chain problems: long waiting times
Persuasion		
	• Insufficient training and education of providers about Chagas disease, its diagnosis and treatment	• Insufficient training and education of providers about Chagas disease, its diagnosis and treatment
	• Lack of political champion for the disease	• Lack of political champion for the disease

*underlined text represents differences in barriers to access between the two drugs*.

#### Regulation

Several regulatory obstacles at the national level were identified. These included regulations regarding drug authorization, medicine importation, and the lack of inclusion of benznidazole and nifurtimox on the national formulary list.

According to Mexican law, all importers of medicines must secure marketing authorization for the products they wish to import [39]. The Federal Commission for Protection against Health Risks (COFEPRIS) is the national medicines regulatory authority and responsible for granting market authorization. Neither benznidazole nor nifurtimox has a market authorization in Mexico and a one-time importation permit must be obtained to import either medicine into Mexico [40].

As both medicines are not marketed in Mexico, they are not included in the national formulary and the institutional formularies including SP formulary (CAUSES) [41].

#### Financing

The regulatory status of both benznidazole and nifurtimox has important implications for financing available to purchase these medicines. Their lack of COFEPRIS authorization and exclusion from the national formulary and the SP formulary precludes the usage of SP medicine procurement funds to purchase them and leaves few avenues for funding [39]. In addition, until 2012, Chagas disease was not included in the package of interventions covered under SP (hereafter CAUSES) for the reason stated above, further limiting financing available to purchase medicines for treating Chagas disease [28].

The financing status of the medicines also affects the clinical quality of treatment because nifurtimox can be procured at no cost through the WHO-Bayer Nifurtimox Donation Program while benznidazole is currently sold on the private market outside Mexico and must be purchased [23], [24].

#### Payment

As noted above, the WHO-Bayer Nifurtimox Donation Program pays the costs for the medication, shipping, and tariffs associated with its delivery. Although the national Program on Onchocerciasis, Leishmaniasis and Chagas Disease within CENAPRECE does not pay these direct costs for delivery to Mexico, there are additional costs associated with drug distribution within Mexico.

#### Organization

Our assessment of organization identified several key challenges to access in this area, including the case registration process, the status of the disease under SP, current Chagas disease treatment guidelines, and problems with the international supply chains for benznidazole and nifurtimox.

As per Figure 1, the case registration process requires that patients receive at least three and sometimes four or more diagnostic tests from two different labs before officially registering a case. Of note, the state is responsible for registering new cases, while the national level is responsible for confirming cases identified at the state level and aggregating data reported by each state on new cases registered. The current WHO diagnostic guidelines recommend that samples be assayed using two distinct tests before a confirmed diagnosis is made [12]. The additional testing sourced from different labs can introduce significant delays and costs between initial diagnosis and treatment in Mexico.

In terms of general policy guidance on the management of Chagas disease, the Mexican Secretary of Health has established official guidelines for the epidemiologic surveillance, prevention, and control of vector-borne diseases that were updated in 2010 [42], [43]. The 2010 policy guidelines for vector borne diseases state that nifurtimox is the first-line antitrypanosomal therapy in patients (up to 70 years of age) who have either acute or indeterminate Chagas disease [43]. Comparison with the 2002 policy guidelines for vector borne diseases demonstrates that the indications for antitrypanosomal therapy have been expanded to include consideration of treatment in adults in the indeterminate phase, as is consistent with the most recent scientific evidence [9], [17], [42]. Though the most recent policy guidance provides general support for the use of antitrypanosomal treatment, Mexico's national clinical guidelines database (CENETEC) for physicians to use when treating patients do not provide instructions on treatment of Chagas disease. Furthermore, neither medicine is included on the Mexican essential medicines list, though they are both included on the WHO Essential Medicines List [44]. In 2012, Chagas disease was added to the SP CAUSES under the group of “Remaining Infectious Diseases” [41], a category that includes leishmaniasis and rickettsia, amongst others. While the addition of Chagas disease to the CAUSES represents an important step forward, it does not include any clinical description of the disease course, nor does it name nifurtimox or benznidazole as treatments [41].

Finally, supply chain problems for benznidazole and nifurtimox that were reported for Mexico included long waiting times of 4 months to more than 2 years to procure either medicine from sources outside the country, and a long, complex application process to secure the necessary COFEPRIS importation permits [45].

#### Persuasion

The primary challenge that was reported related to the persuasion area is a lack of understanding and awareness of the disease, its diagnosis, and treatment among health professionals and populations at risk. Although the Secretary of Health provides physician and health worker trainings about Chagas disease and some health education activities for the general public [38], multiple key informants mentioned the lack of awareness and understanding of Chagas disease by physicians, health workers and the general population as well as fears or misconceptions about the use of antitrypanosomal medicines among physicians as additional obstacles to increasing diagnosis and treatment of Chagas disease in Mexico. Existing evidence has shown a lack of understanding of the disease among the general population physicians and health workers. For example, studies in Morelos show that a substantial proportion (about 45%) of the state population is aware of the triatomine vectors that transmit *T. cruzi*, but few (about 15%) have an understanding of the disease, its clinical consequences and how to prevent and treat it [46], [47]. Because both nifurtimox and benznidazole are associated with adverse side effects, physician and health worker concerns about treatment are not uncommon [18], [20], [48]. Of note, the design and implementation of education programs is a responsibility of the state Secretaries of Health; as such there is substantial heterogeneity in these programs in different states.

#### Other challenges: National prioritization

In addition to these challenges, controversy has existed for several years regarding estimates of the burden of Chagas disease in Mexico. While epidemiologic studies estimate that the national prevalence could be as high as 1.6% [49], [50], [51], important actors, including some officials in the Secretary of Health, have stated in public documents that data from the national blood bank indicate Mexico has a much lower estimated national prevalence of 0.65% or approximately 733,000 cases [52], and that Chagas disease remains a focal problem that does not warrant significant national attention [36]. These public statements highlight important discord about the extent to which both health resources and policy attention should be allocated to the control and treatment of the disease.

## Discussion

This study provides evidence regarding the extent of treatment access for Chagas disease in Mexico and the barriers that influence the level of access. In particular, the study demonstrates that the number of Chagas disease cases registered at the national level in Mexico since 2007 is approximately 0.41% of expected cases and that 120 registered, eligible cases were awaiting treatment at the time of the study. These findings also indicate that Mexico has made an effort to register new cases and provide treatment at both the state and national level and thus show an increased commitment to addressing this disease in Mexico.

Our findings also demonstrate that epidemiologic surveillance for Chagas disease remains a challenge in Mexico and that the complexity of the case registration system may delay or limit registration. Evidence from national data shows that problems in the supply chain of medicines make it difficult to ensure timely access to treatment as cases are registered and further, that the medicine provided by the national program since 2009 has exclusively been nifurtimox, a medicine that has been identified in the clinical literature and international guidelines as second-line therapy [20].

The lack of awareness and understanding of the disease and its treatment among both physicians and populations at risk was another important challenge related to the persuasion policy intervention area [46]. Patient and provider awareness of the disease has implications for efforts to strengthen epidemiologic surveillance and the willingness of physicians to treat infected patients when medicines are available. Additionally, access to treatment for Chagas disease has until 2012 been further weakened by its exclusion from the package of health interventions that are covered under SP [27], [28]. While its addition to the CAUSES in 2012 represents an important step (organization) toward increasing access to treatment, clinical information about the disease is still lacking in this document and neither benznidazole nor nifurtimox is listed as a treatment for the diseases in this category.

In addition to these barriers, it is important to acknowledge the role of international actors and policies as barriers to access to treatment for Chagas disease in Mexico and potentially in other countries as well. The global shortage of benznidazole in 2011 and the challenges in obtaining nifurtimox through WHO exist outside the Mexican context but directly affect efforts by the Mexican national and state control programs to increase access to treatment [23], [24].

These findings provide new information on the state of treatment for Chagas disease in Mexico and the barriers that prevent more widespread access. Previous work on this subject has suggested that efforts to control and treat Chagas disease in Mexico are insufficient [36], [53] but no study has previously measured the gap in access to treatment or analyzed related obstacles. In addition, a recent study estimated the economic burden associated with Chagas disease to exceed seven billion dollars globally and several studies have described the need for increased treatment globally [3], [5], [33], [53], [54]. This study is one of the first to examine the multiple complex factors within the health system that prevent more widespread treatment access in a particular country setting. It is important to note, however, that the state of Morelos did successfully procure benznidazole and offers an important case for showing how a state can take significant initiative in improving access to treatment for Chagas disease.

Some of the findings from the Mexican experience may be relevant to treatment access for Chagas disease in other countries in the region. For instance, reliance on nifurtimox as a first-line therapy in both the 2010 Mexican guidelines for vector-borne diseases and in procurement of medicines at the national level raises questions about the reasons for this choice and whether other countries may also choose to procure nifurtimox through the donation program now or in the future instead of purchasing benznidazole through the private market. In the case of Mexico, the regulatory status of the drugs, especially the lack of commercial permits for them, and the exclusion of antitrypanosomal therapies for Chagas disease from the Mexican national formulary have severely limited sources of financing to buy benznidazole, causing the national program to instead rely on the free nifurtimox. However, little information exists about whether other countries also rely on nifurtimox as a first-line therapy and if so, why. Though clinical guidelines overwhelmingly suggest that benznidazole is better tolerated and that the clinical evidence of its efficacy is more robust, clear international consensus guidelines for the treatment of Chagas disease have not been published and relatively limited data are available about the use and clinical outcomes for the two drugs by different countries around the world.

There are several limitations to this study. First, data on the prevalence of Chagas disease are limited both in Mexico and globally. This constitutes an important challenge to efforts to address this disease in Mexico. In this analysis, we use the official 2010 prevalence estimate from the Mexican Secretary of Health because it is more conservative than the most recent WHO estimate and because the WHO estimate does not have a clear evidence base. This choice may result in our analysis showing greater access to treatment (as a proportion of total infected cases) than may actually exist in Mexico. Some actors within the Mexican Secretary of Health have argued that the epidemiology of Chagas disease in Mexico is focal and that states with a high burden of disease should undertake activities to address this disease at a state level, while others have maintained that the prevalence of Chagas disease is substantial across much of the country and that the disease should be a national priority, especially given the migration of populations from endemic areas both within Mexico and from neighboring countries to Mexico [36], [50]. To provide a more reliable estimate of national prevalence, a nationally representative epidemiologic survey could be conducted, both nationally and by state. This would advance efforts by both the state and national programs to make more informed decisions about the priority and resources that are warranted for Chagas disease treatment.

A second limitation is that we consider benznidazole as the first line antitrypanosomal medicine, despite the lack of definitive international consensus on this issue. We made this decision because benznidazole is being used exclusively as the reference treatment regimen in clinical trials of new drugs, is named as the first line therapy in the treatment guidelines of several non-governmental organizations [20], and is cited as such in the vast majority of the clinical literature [9], [17], [18]. It is worth noting, however, that there is some diversity on treatment regimens within Mexico. Although the national program has used nifurtimox from the WHO donation program, the state of Morelos in Mexico has purchased benznidazole for its treatment program. Morelos registered 263 cases between 2007 and 2011, and treated 148 cases with benznidazole and 4 with nifurtimox.

This study was also limited by lack of data availability at the national and global levels. At the national level in Mexico, this included a lack of national treatment guidelines or data prior to 2010, a dearth of information about treatment eligibility or patient refusal of treatment, and a lack of data on treatment dose, completion or clinical outcomes. In particular, it was difficult to determine what proportion of patients would be treatment eligible according to the guidelines given that no data were available on co-morbidities or patient clinical history that would allow a more thorough analysis of patients in whom treatment may be contraindicated. Furthermore, there is limited evidence about access to treatment in other countries to provide a comparison for assessing Mexico's achievement in this area. Of note, however, a recent study estimated that less than 1% of those infected with *T. cruzi* receive treatment globally, suggesting that the extent of access in Mexico is likely to be similar in other countries [5].

Based on these findings, there are three important strategies that could be undertaken to increase access to treatment for Chagas disease in Mexico.

First, under regulation, an effort could be made to ease the importation process for these drugs. Ideally, this could be accomplished by securing COFEPRIS approval for both medicines and adding them to the national formulary, which could require actions by the relevant producers of benznidazole and nifurtimox. However, as noted above, benznidazole and nifurtimox are not approved by the United States Federal Drug Administration or the European Medicines Agency, in part because full clinical trials have not been completed for either drug. This lack of approval from two leading regulatory bodies may affect the willingness of other national regulatory bodies to approve the medicines. That said, both medications are included on the WHO Essential Medicine List [55]. In addition, clinical evidence continues to accumulate in favor of these drugs and efforts by institutions such as the Drugs for Neglected Diseases Initiative are being made to register the drugs in countries such as Colombia, Paraguay and Bolivia. In other contexts, alternative regulatory approaches such as investigational protocols are being utilized to make the drugs available [18]. Also with respect to regulation, countries with a high burden of Chagas disease may consider instituting laws that mandate rigorous epidemiologic surveillance and health education as well as prevention, diagnosis and treatment of the disease. For instance, Argentina offers a model for such legislation in National Law No. 26281. This law requires, among other things mandatory diagnostic testing and reporting for Chagas disease in all pregnant women and in newborns in the first year of life born to infected mothers.

Second, under persuasion, efforts could be expanded to provide disease-specific health education programs on Chagas disease for physicians, healthcare providers and populations at risk. Increased awareness of the disease and a better understanding of appropriate treatment methods is a critical aspect of strengthening case registration and access to treatment. In addition, health education activities have been emphasized in other national control programs such as those in Guatemala [56] and the Southern Cone initiative and have been used alongside vector control to increase awareness of the disease in high risk communities and among physicians and health workers. Increased awareness of the disease and of treatment methods is a critical aspect of strengthening case registration and access to treatment. Given the importance of this programming, the WHO and PAHO also play a potentially important role in terms of encouraging these programs and providing guidance on their design and implementation.

Third, under organization, it is important to strengthen existing guidelines in Mexico for the diagnosis and treatment of Chagas disease and information availability about the supply chains for these two medicines. This includes the addition of a clinical description of Chagas disease and the two medicines to its entry in the CAUSES and the creation of a clinical guide for diagnosis and treatment as this information is critically important to strengthen awareness of treatment for Chagas disease and information for practitioners about how to diagnose and treat the disease. In addition, better public reporting of medicines released and used at the state, national and global levels is needed.

In conclusion, this study found that access to treatment for Chagas disease in one high burden country (Mexico) is limited in important ways and identified three critical obstacles to treatment access: regulatory barriers to importation, a lack of understanding of the disease and its treatment, and a dearth of clinical guidelines [5]. Several of these barriers are likely to affect access in other countries as well, especially the lack of regulatory approval and registration of benznidazole and nifurtimox and the lack of publically available information on their supply chains. Finally, the study proposed a series of actions that could be taken in Mexico, based on a general analytical framework, to improve access to treatment for Chagas disease. These recommendations have important implications for other countries in the region with similar problems in access to treatment for Chagas disease.
